# Sting Stories: Firsthand Experiences of Fish Envenomation Through a Small-Scale Questionnaire

**DOI:** 10.3390/toxins17030134

**Published:** 2025-03-13

**Authors:** Richard J. Harris, Silvia L. Saggiomo, Gillian Paxton, Cherie A. Motti

**Affiliations:** 1 Australian Institute of Marine Science (AIMS), Cape Cleveland, Townsville, QLD 4810, Australia; c.motti@aims.gov.au; 2 Queensland Institute of Medical Research Berghofer (QIMR Berghofer), Brisbane, QLD 4006, Australia; silvia.saggiomo@qimrb.edu.au; 3 The Cairns Institute, James Cook University (JCU), Cairns, QLD 4870, Australia; gillian.paxton@jcu.edu.au

**Keywords:** fish venom, fish sting, hot water immersion, survey, pain

## Abstract

Stings from venomous bony and cartilaginous fishes are known to cause extreme pain in humans, and with changing migratory patterns and distributions due to climate change, human interactions with venomous fishes may increase. Therefore, developing a better understanding of venomous fish stings and the associated pain can provide better solutions for first aid and treatments, particularly in areas or within populations with a higher risk of being stung. Using the results from an online questionnaire, this study discusses the perspectives of 121 people with direct experience of fish stings, exploring the contexts in which fish stings occurred, their firsthand experiences of pain, sting pathophysiology, experiences with medical treatments, and the long-term consequences of fish stings. This small-scale survey has proved successful for the exploration of fish sting experiences, and as such, an approach of this nature should be considered to better understand victim’s experiences with other painful animal stings.

## 1. Introduction

The defensive stings caused by venomous bony and cartilaginous fishes are algogenic and are anecdotally described as some of the most painful animal-human experiences. One victim of the notorious stonefish (*Synanceia* spp.) reported, “I describe the pain as excruciating because the word comes from crucify, and that’s exactly what it was—there’s [sic] was no other way to describe it” [1]. The defensive utilisation of venom in fishes is achieved through toxins that elicit pain (through direct and/or indirect pathways) in conjunction with sharp spines for the most effective delivery into a biological system [2,3,4].

The pain is often described as being disproportionate in intensity to the mechanical damage caused by the spines [5]. Pain-producing defensive venoms, by their nature, do not need to cause death or incapacitation but merely need to be effective in immediately discouraging the predator (or opposing organism) from attacking so as to increase the defending organism’s escape potential and survival [6,7,8]. In this scenario, causing extreme localised pain is an effective method as both a short-(instant survival effect) and long-term (predator learned avoidance) defence strategy [7,9,10].

Humans are not the intended target for which fish venoms have evolved; however, due to painful defensive toxins often having target ubiquity or pharmacological promiscuity [8], they can be extremely effective against humans by proxy. Aside from the intense pain, other symptoms and pathologies, both local and systemic, often arise, including allergic reactions, cardiac arrhythmia, dermonecrosis, erythema, excessive bleeding, hypotension, infection, oedema, and vomiting [11,12,13,14].

The medical treatment of fish stings is mostly focused on reducing the pain until the symptoms subside. The primary recommended medical treatment to ease the pain following marine stings is hot water immersion (HWI) therapy [15], which involves submerging the affected limb in hot water at ~45 °C [16] for 90 min [13,17,18,19,20,21]. However, little is understood as to how or why the pain sensation seemingly dissipates with HWI [2,22,23]. Other medical treatments for analgesia include anaesthesia (inhaled or local), antibiotics, antivenom (when available), bleeding control, corticosteroids, nonsteroidal anti-inflammatory drugs (NSAIDs), tetanus prevention injections, peripheral nerve blockade, and surgery to either remove embedded or dislodged barbs and spine debris or to treat secondary issues, such as necrotising fasciitis [17,24,25,26].

Despite the fact that venomous fish stings are extremely painful to humans, there have been very few studies that have explored the personal experiences of being stung. A recent study used a survey to explore the pain perception of Red Lionfish (*Pterois volitans*) stings [27]; however, the sole focus was specifically on pain experience and how the pain affected people’s lives. While this study provided much needed foundational insights into painful fish stings, it did not explore other aspects of envenomation, such as types of treatment received and how effective such treatments were at alleviating pain. Further, it was solely focused on a single species. Therefore, there are large gaps in our understanding of how victims experience not only pain but also medical treatments and personal ordeals in response to fish stings across many different venomous species.

Rising global water temperatures are altering animal migration patterns and distributions [28], aiding in the spread of invasive fish species [29] and increasing the likelihood of venomous fishes entering new regions, particularly popular tourist destinations, thereby increasing the risk of stings [28,30]. Fish envenomations are already the biggest culprit in stings caused by marine organisms [31], particularly in fishing communities [32,33]. Due to the increased likelihood of human-fish interactions, there is a need to extend such surveys to include other venomous fish species. Therefore, a better understanding of how and why stings occur, which species are more likely to cause medical problems, and how best to treat and manage stings is imperative in a rapidly changing aquatic environment.

Here, a small-scale survey approach was applied, building on the previous *P. volitans* study [27], using an online questionnaire format to invite personal insights from people with experience of venomous fish stings. As part of this survey, participants were asked to provide information about themselves and a written description of the fish sting incident, including the fish species involved, the context in which the sting occurred, and their experiences of pain. Our aim is to compile comprehensive data from firsthand accounts of fish envenomation to enhance the knowledge base for volunteers and practitioners, thereby improving first aid strategies and clinical management.

## 2. Results and Discussion

### 2.1. Envenomation Incidences

**Figure 1:**
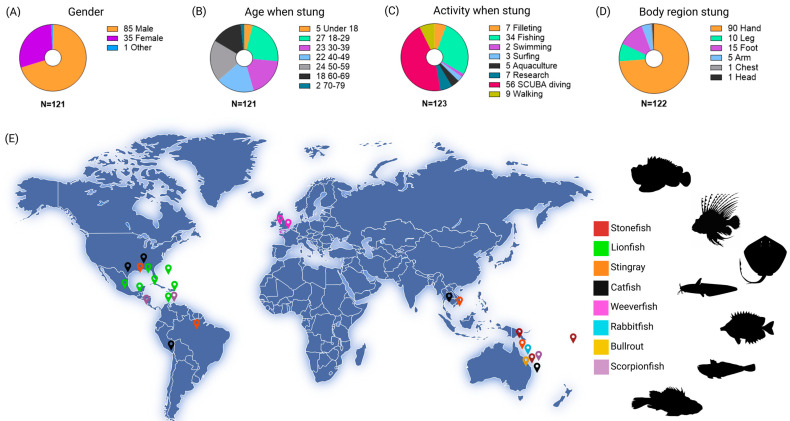
Summary of research respondents showing the following: (**A**) gender distribution of participants; (**B**) age of participants when they were stung; (**C**) activity the participant was engaged in when stung; (**D**) body region where the participant was stung; (**E**) global distribution of fish sting occurrences, colour-coded by the venomous fish species reported. Some data points on the map encompass multiple participant data that overlapped, and pin locations are close approximations based upon both participant data and known distributions of fish species. N values that are greater than the number of participants (121) refer to where respondents reported data for multiple stings. Figure was created in Biorender, and fish images were taken from Phylopic under CC PDM1.0.

A total of 121 people responded to the survey (Figure 1), where 72 of those participants reported a single incidence and 49 participants reported more than one incidence. Respondents reported being stung in 31 different countries around the world (Supplementary Data S1).

While demographic information about participants (gender, age, and sting location) was collected and shown in Figure 1, it is not the purpose of this exploratory research to demonstrate generalisable demographic patterns in fish stings. The majority of survey respondents were male (85 respondents) (Figure 1A) and between 18 and 59 years old when stung (Figure 1B). People aged under 18 and 70–79+ were the least represented age categories in the study (5 and 2 individuals, respectively; Figure 1B). The majority (97) of fish stings were reported around the Americas (USA, Central and South America, and Caribbean islands), followed by Australia (27) (Supplementary Data S1).

Survey participants were engaging in a range of activities when stung, including swimming (2), surfing (3), and aquaculture (5; Figure 1C). However, the majority were engaged in SCUBA diving (56) or fishing (34). While this aligns with existing research recognising higher risks of marine stings amongst fishers [34], it also indicates that SCUBA diving may carry an increased risk of fish stings. However, the increased risk of being stung while SCUBA diving within the respondent cohort is likely associated with the culling of lionfish (*Pterois* spp.). This is evidenced by the frequency of SCUBA-related envenomation incidents involving lionfish (54 of the 56 incidences reported) and the common practice of culling these invasive species using SCUBA gear in affected regions [29,35,36]. In relation to the most common body regions stung, our respondents reported the hand as the most commonly stung site (90; Figure 1D), followed by the foot (15). This suggests that activities involving the handling of fish—whether during culling, fishing, cooking, or other contexts—is also a risk factor for fish stings. This is consistent with the association observed above, between fishing and SCUBA activities and fish stings. Culling lionfish requires spearing and subsequent manual removal of the carcass from the spear, and fishing requires the manual removal of fish from hooks or nets.

The most common fish species involved in stings were the lionfishes (67), catfishes (16), and stingrays (14). Other incidences were caused by bullrouts, rabbitfishes, scorpionfishes, stonefishes, and weeverfishes (Figure 1E).

### 2.2. Experiences of Pain

Participants were asked to evaluate the pain from envenomation according to a 0–10 scale where 0 = no pain and 10 = worst pain imaginable [27]. Overall, participants reported experiencing moderate-to-high levels of pain immediately after being stung and up to 2 h later, with a distinct peak in pain rating at 30 min, followed by a decrease in intensity from 1 h onwards (Figures S1 and S2). The vast majority of pain ratings dropped to below 0.8 after 48 h and 0.4 after 1 week (Figures S1 and S2), suggesting that, without complications arising from other medical conditions, the pain considerably subsides after 1 week. The pain data gathered here, across all species and groups, are also consistent with [27], which similarly found pain peaks between 30 min and 1 h after the sting, then steadily decreases thereafter until the pain dissipated within a week (with some exceptions).

When asked to describe the type of pain associated with fish stings, 88 respondents reported pain that was ‘hot and searing’ (Table S2). This pain quality has been described in association with Red Lionfish [27] and Bullrout [37] stings. A smaller number of respondents used descriptors such as an ‘electric shock’ (4), ‘pins and needles’ (6), ‘itchiness’ (3), and ‘throbbing’ (4).

No difference was observed between how male and female respondents rated their experiences of pain in this study (*p* > 0.05; Figure S3), consistent with reports of gender comparisons in pain ratings of *P. volitans* stings [27]. For the majority of the age group categories (18-69), there was also no significance found in pain perception over time within this cohort (*p* > 0.05; Figure S4). Due to the under 18 and 70–79+ age group data having a small number of participants (5 and 2, respectively), a statistical analysis with those data is unreliable.

**Figure 2:**
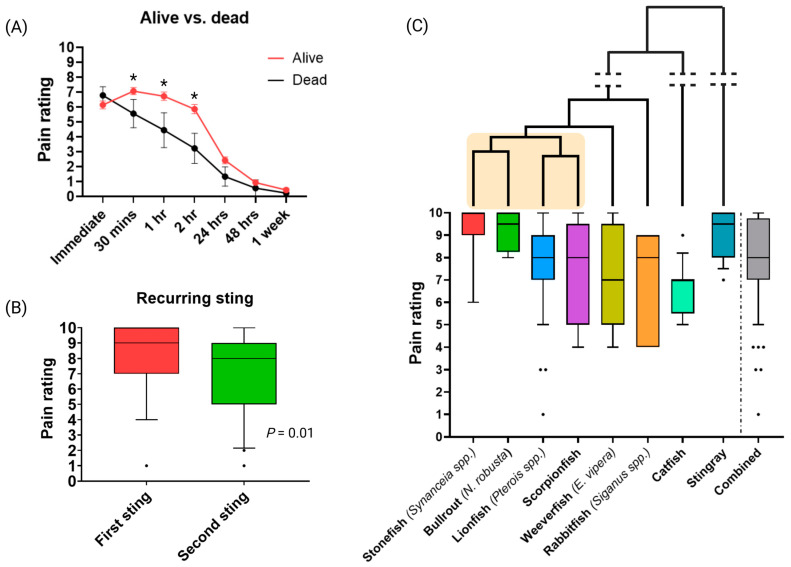
Differences in self-reported pain experiences relating to fish stings: (**A**) Pain induced by stings from living vs dead fish. The * reports a significance of *p* > 0.05. (**B**) Reports of pain in subsequent stings by the same species of fish (N = 42). (**C**) Relationship between fish phylogeny and reported sting pain levels. Each box plot represents the data distribution, with the dark line in the centre of the plot showing the mean maximum pain rating according to each fish species and group. Error bars represent Standard Deviation (SD). Black dots represent data outliers based on a 5–95 percentile limit. The shaded box indicates the scorpaeniformes grouping.

Unsurprisingly, stings from living vs dead fish resulted in a significant difference in pain rating (*p* > 0.05), where living fish induced a higher pain rating over a longer period of time (i.e., immediate to 2 h) compared to dead fish, where there was a steady decline following the sting (Figure 2A and Figure S5). However, by 24 h, the pain score showed no significant difference between the two cohorts, with pain levels further declining up to 1 week after the sting (Figure S5). This is consistent with findings of pain perception in *P. volitans* stings whereby living lionfish induced a higher pain rating score [27]. It is likely that once the fish dies, biochemical maintenance and processes that stabilise the components within the venom cease, and degradation of proteinaceous toxins begins shortly after death, thereby ultimately reducing the pain intensity. The labile nature of some fish venom toxins (e.g., cytolysins; CTx) has been demonstrated under laboratory conditions [38,39,40]. However, it must be noted that not all toxins are likely to be degraded, that stings from dead fish should be treated as seriously as stings from living fish, and that victims should still seek medical care.

Forty-two participants reported being stung by the same species on more than one occasion. Within this cohort, a significant difference (*p* = 0.01) was observed in the ways pain was rated between the first and subsequent stings (Figure 2B and Table S3), with most reporting a lower pain rating for the subsequent sting. This interesting observation may be consistent with insights gained in the *P. volitans* study, where lower pain levels were reported by sting victims after the initial 2 h when stung for the second time. There may be several reasons for this, including psychological effects, such as the reduction in fear of the pain and symptoms due to knowledge of treatment effectiveness [27] and immunological effects, where the body develops specific antibodies against some fish venom toxins [41,42]. The generation of antibodies requires time (days to weeks) to become fully active, but this adaptive response provides toxin-specific and long-lasting protection for the victim, making any pain induced by an immunological response to a future sting more effective and efficient, particularly if the victim was stung by the same fish species, as was the case with many of our respondents. However, this would only account for toxins that cause pain through an immunological response. So, being stung by a venomous fish may help the victim in subsequent fish envenomations by reducing the pain induced by a select group of toxins; however, given the nature of painful cytolytic defensive venoms, it would seem unlikely that humans would develop complete resistance or conditioning, particularly from infrequently recurring stings. More research is needed in this area to decouple the physical, emotional, neurological, and immunological mechanisms that determine pain perception.

### 2.3. Pain and Fish Species

Stonefish (*Synanceia* spp.) and the closely related Bullrout (*Notesthes robusta*—also referred to as the Freshwater Stonefish) were given the highest average pain rating (9.2 out of 10) by those respondents who had experienced these stings (6 and 4 respondents, respectively) (Figure 2C). This is unsurprising given both species are notorious across Australia for their painful sting [43,44,45]. However, despite their close phylogenetic relationship (Figure 2C), preliminary investigations indicate the predominant pain-causing toxin from *N. robusta* does not seem to conform to the CTx family of toxins prevalent in venomous scorpaeniformes [46,47]. This is also evidenced by the fact that *Synanceia* antivenom does not cross-react with *N. robusta* venom components [44,46]. The pain-causing toxins within *N. robusta* venom remain to be fully investigated, as does their molecular evolutionary divergence. Further evolutionary research on *N. robusta* venom is warranted to develop more effective targeted first aid and pain treatments and to understand the physiological targets of the venom toxins.

The 14 participants who reported being stung by a stingray gave these stings an average pain rating of 9, making this pain similar to that reported in incidents involving stonefish or Bullrouts. Given their unique venom apparatus, having retroserrated detachable barbs that can be deeply embedded and which allow for maximal efficiency of toxin diffusion [2], it is unsurprising that the pain perception reported by this cohort is also extremely high. Deaths resulting from stingray stings, mostly from the mechanical damage to vital organs [48,49,50,51], along with statistics indicating stingrays cause the most marine animal-related injuries [31,52], have raised awareness in the general community, yet stingray venoms still remain understudied [2,3], and with severe side effects including cardiovascular failure, research to elucidate the chemical nature of the toxins is critical for improving clinical outcomes of stingray envenomation and developing drugs to target the underlying pathophysiology.

The pain experiences reported here seem consistent with other research [27] where it was also found participants reporting *Pterois* spp. stings with an average pain rating of 7.2 (±2.69 vs what was reported here: 6.9 (±2.49). This not only confirms the robustness of the survey methodology across studies but also provides confidence that the reporting on stings caused by other fish species is reliable. It is envisaged that the expansion of this survey to a larger sample size will provide an informative database for clinicians and first aid responders, as well as provide more informed data that can give insights into future avenues on venom biochemistry and the evolution of fish venom systems.

### 2.4. Sting Pathophysiology

All other non-pain-associated sequelae that arise from both the toxins and the mechanical damage of the sting likely provide further defensive factors that are necessary for successful deterrence and for long-term operant conditioning of the predator [9,53]. Understanding the likely pathophysiological consequences that arise may help clinicians be better prepared for treating fish stings.

Redness (reported by 81 respondents) and swelling (79) were the most common symptoms reported by participants (Table S4), consistent with clinical studies [12], followed by necrosis (11) [11,12,17]. Early necrosis is likely to occur due to cytotoxic components of the venom that promote bacterial infection [54] and (dermo)necrotic activity. Further commentary is discussed in the following section regarding bacterial necrosis and subsequent treatments.

Secondary infections of fish stings are identified as clinical manifestations, with some extreme cases leading to toes and even the forearm being amputated following necrotising fasciitis and septic shock [17,51,55,56,57,58]. Overall, six participants in this study reported infection (Figure S6). One respondent experienced cellulitis, which has also been reported as a secondary problem in fish envenomation [11,57,58] and was treated in hospital for three weeks. Some authors have posited that there may be components within stingray (and possibly other fish) venom that interrupt the haemostatic system and, by proxy, promote bacterial growth and subsequent infection at the wound site [59].

Six participants also noted experiencing allergic reactions to stings (Figure S6). Allergic reactions to fish stings may be a sign of hypersensitivity to fish skin mucin glycoproteins that bind Immunoglobulin E (IgE) [60]. One participant noted that after their first sting, they “developed a sensitivity to lionfish stings and even the fish slime [skin mucus]”. Given that the current hypotheses for fish venom evolution are centred around the recruitment of skin surface/mucus toxins [2], it would not be surprising if some venom components can elicit an immunological allergic response similar to that of skin mucus, inducing long-term hypersensitivity, as is seen with many insect venoms [61,62]. It might also be that participants are attributing a self-diagnosed clinical condition that has not been officially analysed by a clinician, and therefore, their definition of an allergic reaction might not align with official clinical assessments.

Although not reported in this study, other clinical manifestations, such as Reynaud’s phenomenon [63] and compartment syndrome [64], have also been reported in some fish envenomations. In one extreme case, a pregnant victim had a spontaneous miscarriage after a fish sting [65]. However, it remains unknown whether the sting was the direct causation. Therefore, it is clear that the direct impact of fish stings transcends the simple inconvenience of a short-lived painful sting.

Given the understudied nature of fish venom toxins and their clinical manifestations [2,3,4], it is difficult to ascertain which toxins are the most likely candidates for causing these pathophysiological complications. Therefore, it is imperative that future research bridges the gap between the clinical effects of the venom and the underlying toxin functionalities, thus giving a clearer picture of the connection between toxin composition and the clinical outcomes of stings.

### 2.5. Hot Water Immersion Therapy and Other Treatments

There is a wealth of evidence to support the use of HWI in non-life-threatening cases of painful marine stings (see reviews such as [15]), some of which focus solely on fish envenomation [20,56,66]. The overwhelming majority of the data suggest that pain is alleviated with the use of proper HWI therapy, and it is the recommended immediate treatment. Here, half of our respondents (61 of our 121 respondents) reported using HWI to treat their fish stings.

**Figure 3:**
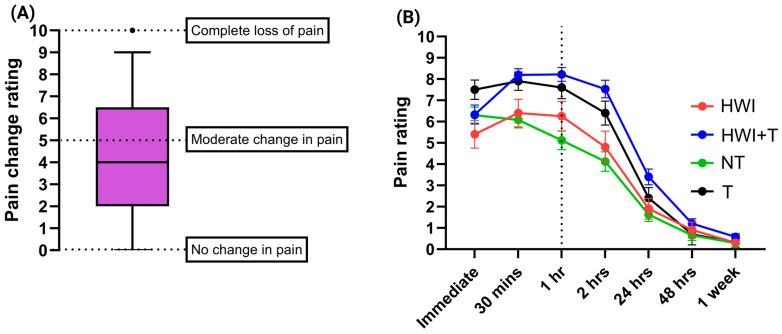
Effectiveness of Hot Water Immersion (HWI) therapy in reducing pain: (**A**) The pain change rating when the affected area was submerged. Moreover, 0 indicates no change in pain, and 10 indicates a complete loss of pain. The black dot above the error bar indicates a likely outlier data point based on the 95% confidence cut-off limit. (**B**) The pain rating over time across the different treatment groups. No treatment = NT; Hot Water Immersion Therapy treatment only = HWI; pain treatments only = T; HWI with other treatments = HWI + T. All error bars indicate Standard Deviation (SD). The dashed vertical line at 1 hr is indicative of the approximate most common time that any form of treatment was first applied (see Supplementary Raw Data).

Of the 61 people who received HWI as a first aid treatment, 47 participants reported an alleviation of the pain (Table 1). This appears to be consistent with previous studies that determined HWI to be an effective treatment to relieve pain in 56–100% of sting cases [17,56,67,68]. According to the pain change rating data (Figure 3A), HWI seems to reduce a moderate amount of pain (i.e., pain change rating average of 4 out of 10) when the affected limb is submerged. This too is in accordance with previous studies that have shown pain to be reduced rather than completely diminished [17,20,67,68,69]. Here, some participants provided testimonies such as, “Hot water immersion was of great benefit”, “With the hot water on [the affected limb] there was almost no pain. Without hot water the pain was bad”. Again, these data are consistent with the literature confirming the effectiveness of HWI in treating fish envenomations [13,20,21,68,70].

Assessment of the effectiveness of HWI against other treatment combinations (only pain medication such as anaesthetics and pain killers were assessed; Table S5) in reducing pain over time (Figure S7) showed a statistically significant difference in pain change (1 h vs. 1 week) between no treatment (NT) vs. HWI with treatment (HWI+T) or no HWI but with treatment (T) (*p* < 0.01) (Figure S7) However, surprisingly, there was seemingly no statistically significant difference between no treatment (NT) vs. HWI only (HWI) (*p* > 0.05) which is contradictory to the responses given by the 47 participants who noted a reduction in pain when the affected area was submerged (Table 1). This suggests that although some of the respondents in this study feel immediate pain relief with HWI, the overall pain reduction profile mirrors that of the no treatment, i.e., pain does not diminish at a faster rate over time with HWI. This might also be why the simultaneous application of other forms of pain medication (Table S5) that directly target nociceptive pathways, i.e., those activated by tissue damage induced by the penetration of the spine/barb (49 respondents receiving nonsteroidal anti-inflammatory drugs (NSAIDs) and 10 receiving anaesthetics—local/inhalation, both designed to decrease the sensitivity of nociceptors), were able to achieve faster relief.

Overall, the data suggest that for the respondents within this study, the most effective and fastest pain-relieving therapy is HWI combined with other forms of pain treatments (HWI+T; Figure 3B and Figure S7) and is reflective of the outcomes of another study [69]. Further, when HWI is not available, then pain medication alone helped reduce the pain for our respondents (Figure 3B and Figure S7).

However, there are some things to note regarding the data. Firstly, the peak pain level for those given pain medications (HWI + T or T) is higher than those that only received HWI or NT. This might imply that the reason why those respondents received additional pain treatments could be because the pain was more intolerable than the other groups. Further, those that received treatments (HWI, HWI + T, or T) all reported an increase in pain level, which evidently then required pain relief, whereas, in the NT group, the pain level decreased and continued to do so, so pain relief was likely not needed.

It has long been hypothesised that the temperature of HWI aids in toxin deactivation due to many fish venom toxins being notoriously heat labile [71,72,73]. However, some authors have debated this hypothesis [22,23]. There are three main points of contention: (i) the temperature needed to denature toxins once they have been infused deep into subcutaneous and/or intramuscular tissue would need to be in excess of the effective 45 °C, likely requiring temperatures that would cause more severe thermal damage to the skin [23,74]; (ii) pain relief with HWI can be instantaneous upon submersing the limb, this timeframe being too rapid for the biochemical inactivation of all toxins [23]; (iii) removal of the affected area from hot water causes the pain to return [12,23,55,74].

Alternative mechanisms for why HWI relieves pain have been proposed, such as Gating Control (GC) and/or Diffuse Noxious Inhibitory Control (DNIC) theories of pain relief [23]. The GC theory proposes that stimulation of non-nociceptive fibres can inhibit signals being processed by nociceptive fibres [75]. In the context of HWI, the detection of a temperature change in stimuli would interfere with the sensation of pain. Similarly, DNIC (sometimes also referred to as heterotopic noxious conditioning stimulation, endogenous analgesia system, or DNIC-like effects [76]) functions when nociceptive information is inhibited by noxious stimuli (such as heat or pressure). However, by adding artificial conditions or simultaneously applying multiple/different noxious stimuli, DNIC can suppress or alter nociception [77]. The use of additional stimuli, such as hot or cold, has been shown to significantly decrease pain perception in humans [78]. In this context, it could be why pain from marine envenomations can also be relieved with the use of ice/cold treatments [15,55,79], with some studies finding no statistical significance between HWI vs Icepack treatments [79]. Cold temperatures would also not be effective in denaturing toxins, yet pain sensation is still diminished in many case reports. Further, the application of heat has also been shown to reduce pain caused by other organisms whose toxins are not typically considered to be heat labile, including echinoderms [80,81], scorpions [82,83,84], centipedes [85], and insects [82,86], further adding dispute to the HWI toxin denaturing hypothesis.

Collectively, the findings here lend some support to the alternative hypotheses of HWI pain relief. Further to this, the 32 respondents who received HWI reported that removal of the affected limb from hot water caused the pain to return (Table 1). One participant reported that their “pain went from 7 to 3 instantly” once placed in hot water but went “back up to 7” immediately upon taking their limb out of the hot water. This account appears consistent with previous studies [12,23,55,74] and argues against the toxin deactivation hypothesis. However, due to the complexity of envenomation injuries, it is too early to discredit the HWI toxin deactivation theory. More careful examination of the precise mechanisms that underpin the effectiveness of HWI in reducing pain caused by fish envenomations is needed to improve treatments for these and other animal stings, with the potential to inspire new drug technologies to treat ailments associated with these pathways [87,88,89,90]. 

While it is the recommended treatment for fish stings, consideration should be given to other complications that may arise from the use of HWI. Some studies have suggested that there may be an acceleration of bacterial infection that might induce necrotising fasciitis [91,92,93,94,95]. Because of this, Tang et al. 2006 have urged the need to seek further medical care by a professional with all fish stings, even if pain is diminished [91]. The improper utilisation of HWI can also lead to iatrogenic thermal burns when temperatures are not monitored and become too high (>50 °C) [66,96,97,98]. Here, the majority of respondents who applied HWI treatments used the advised recommended temperature (~45 °C), with only three having burn/scalding complications; two of these were of an unknown temperature and one at 50+ °C (Table S6). These data further support ~45 °C as a safe temperature that is still able to provide significant pain relief.

### 2.6. Ongoing Impacts of Fish Stings

While fish envenomation is not currently seen as a public health concern (cf. snake bites), some authors have posited that they should be treated as a neglected medical problem, particularly in certain regions of the world and across populations who rely on fishing for their livelihood [32,33]. The results of this research indicate that fish stings can have lasting impacts on our research respondents.

Participants reported a range of impacts on general life activities resulting from and lasting up to 1-week following envenomation. The most prominent being impacts on general movement of the affected area (41 respondents), and negative impacts were reported in terms of mobility (7), sleep (9), mood (8), and self-care (22) (Table 2). While only 16 of 121 respondents reported needing to take time off work or education to recover from a fish sting, the time needed could be considerable, ranging from 1 day to up to 3 weeks (Table 2). The respondent who reported requiring up to 3 weeks off also reported severe medical complications, requiring weekly visits to an infection specialist for 6 months following the envenomation incident.

Long-term effects were reported by 28 participants and included permanent loss of sensation in the limb, lack of mobility and strength in the limb, loss of parts of the toe and leg muscle due to necrosis, development of an allergy to subsequent fish stings and fish mucus, itchiness at the wound site, and hardened skin at the wound site (see Supplementary Raw Data).

While fish envenomations may not have the same global or medical impact as venomous snake bites, our data indicate that they can still impact people’s lives. As well as being a very painful experience, being stung by a venomous fish can have long-lasting effects on vocational activities and physical and mental well-being. These impacts and their social implications warrant further attention. For example, it is still unclear what impact experiences of fish stings have vocationally within the marine industries, what systems are in place to protect individuals impacted by fish stings, and the potential strain fish sting incidences create economically and in terms of medical resources. Therefore, it is important to acknowledge that fish envenomations may present a significant medical concern in certain contexts, particularly in high-risk populations, and to advocate for improvements in initial first aid measures and ongoing treatment.

### 2.7. Limitations of the Study

This research was designed to explore the firsthand experiences of a relatively small cohort of people. Its results are not intended to be generalisable to a broader population but provide much needed insights into incidences and effects of fish envenomation.

While recall bias is a common limitation to survey data whereby participants tend to misremember or omit details with regard to their past experiences [99], this is unlikely to be a problem given the exploratory objectives of this research and the descriptive nature of the results. A participant’s self-diagnosis of some symptoms may differ from the clinical diagnosis; e.g., a self-diagnosis of ‘infection’ or ‘allergy’ might not be reflective of what a clinician’s diagnosis is.

The online format of the survey meant it may not have been readily accessible and therefore potentially restricted the number of participants reached, i.e., those who may not have access to the internet, particularly in more remote regions and communities where fishing and free diving are their livelihood. The survey was also further limited in that it was only available in English and not accessible to non-English speaking participants.

This study has a small sample size (particularly in some categories) and deliberately used purposive sampling to gain deep insights from a specific group of people (victims of fish envenomation) that would otherwise be difficult to access using broader statistical survey methods. As an effect of this, the data are not statistically representative. This may be considered a priority for future phases of this research. Examining the broader impacts of fish envenomation, for example, may require a representative sample and the reduction of random variation in the data. Regardless, the consistency of findings here with other aforementioned studies (e.g., [23]) indicates that this study provides important insights and a baseline understanding of fish envenomation against which future studies can be compared.

## 3. Conclusions

These small-scale self-reported survey data exploring multiple facets of people’s experiences with venomous fish stings have provided useful information that can guide improvements in first aid measures.

In summary, the main findings within this cohort of participants were that lionfishes (*Pterois* spp.) were the most common fishes that participants had been stung by and that SCUBA diving was a leading activity where people were stung. Both of these might be correlated with the invasive Lionfish culling efforts. Fishes, such as stonefishes (*Synanceia* spp.), Bullrout (*Nothestes robusta*), and stingrays, produced the highest pain rating amongst our sampled participants, which is consistent with other literature and anecdotal reports. Aside from being a painful experience, fish stings can also cause other medical complications such as infection and necrosis and can have longer term impacts on a victim’s everyday life than previously thought. Amongst our cohort, the most effective pain-relieving treatment was hot water immersion in combination with other types of pain relief medications such as analgesics. Interestingly, although hot water immersion alone reduced the pain experience of our participants, it did not diminish pain any quicker over time compared to receiving no treatment. Therefore, these data might support an alternative hypothesis of how and why hot water immersion works in relieving pain from fish stings.

This research provides foundational evidence for the following: (i) builds upon current knowledge of painful venomous fish stings, which can be applied to other painful stinging organisms. (ii) the use of hot water immersion therapy as an immediate pain relieving strategy (particularly when combined with other pain treatments) is supported; however, the findings do suggest that conventional hypotheses as to why hot water immersion therapy works warrant further investigation.; (iii) venomous fish stings should be considered a greater concern by current medicine based upon reported long-term complications, including physiological, social, and financial. Building upon prior foundational studies that assess pain caused by fish stings, it is recommended that further studies utilise and build upon the survey methodology and questions used here and by [27] to better understand the experiences associated with other painful animal stings.

## 4. Materials and Methods

### 4.1. Survey Design and Distribution

The survey consisted of 51 questions (approx. 10 min completion time). Questions were mostly semi-structured and were designed to elicit a mix of information, including participant demographics and the specific context and outcomes of fish stings while allowing participants the opportunity to share candid detail about their firsthand experience of fish envenomation. The survey automatically redirected participants to relevant questions tailored to their specific answers, so not all participants answered all 51 questions. The survey was delivered using www.surveymonkey.com (accessed on 11 February 2025) and distributed through social media outlets and groups such as Facebook and X (formerly Twitter). Participants were encouraged to further distribute the survey URL on their own social media or directly to people they may know personally who have been stung by a venomous fish.

The identity of all participants remained anonymous and there were no questions posed that could relate to any specific person. Consent was gained at the start of the survey. The raw data (excel file) is stored electronically on an external password-secured hard drive by the lead investigator. Curated data and survey questions can be accessed through the Supplementary Raw Data. The raw data were curated to remove any possible personal identifiers (e.g., if a participant wrote their name in an answer to an open-ended question).

The survey was active for eight months (May 2024–December 2024), with a total of 121 participants providing usable data. During this period, there were some minor live changes to some questions based on participant feedback. These modified questions did not change the outcome of the data. Some specific wordings for questions were amended to remove ambiguity. Also, the survey format was updated to allow participants who had been stung by multiple different fishes to take the survey again.

To ensure consistency and to allow for comparable results, the questions specifically relating to the experience of pain (i.e., pain scale rating) were adapted from the lionfish sting pain experience survey [27], which was originally based on a standardised NIH PROMIS questionnaire (for pain intensity and characteristics) [100]. The pain scale rating was defined from 0 to 10, where 0 indicated no pain, and 10 was the worst imaginable pain. These pain scale questions were assessed over different time points, from immediately after the sting up to one week later.

### 4.2. Ethics

Ethics was assessed and approved by the James Cook University Human Research Ethics Committee under the application ID H9373 on 23 April 2024.

### 4.3. Data Selection and Statistical Analyses

Exclusion criteria for any data included the following: (i) where the participant could not identify what stung them (i.e., simply stating ‘unknown’ with regard to the fish species) or if they stated something that was not a fish, such as jellyfish; (ii) if the fish species they mention is known not to be venomous; (iii) if the questionnaire was incomplete, or the participant skipped important study defining questions; (iv) if the participant was under the age of 18 at the time of taking the survey.

For any data where participants could use their own descriptive keywords or phrases such as in ‘other (please specify)’ type answers, keywords that were matched with pre-existing defined categories were coded to standardise the data. For example, ‘Hot searing pain’ would be coded for if the participant stated a ‘burning sensation’. Similarly, data were curated for answers whereby if an answer of finger or knee was used, these were changed to hand and leg, respectively, to encapsulate the already predefined broader terms.

For hot water immersion therapy and medical treatment data analyses, only treatments that were deemed clinically effective in relieving pain were used within the data. For example, participants who solely answered ‘alcohol’ as a form of pain treatment were not considered as a data point in any ‘treatment’ group. Further, the data analyses only used pain rating data from 1 hr onward since the most common time to receive treatment was in the ‘30–60 min’ and ‘60+ min’ categories.

Statistical analyses and graphs were generated either in GraphPad Prism (v10.2.2) or R console (v4.4.2) using the packages Tidyverse, glmmTMB, emmeans, and DHARMa. A paired t-test was conducted in GraphPad Prism, whilst mixed-effects models were conducted in R. All values are expressed as mean ± SD unless otherwise stated. Differences were considered statistically significant at *p* < 0.05.

All graphical outputs were taken from GraphPad Prism (v10.2.2) and created into final figures in Biorender.com.

## Figures and Tables

**Table 1:** Data describing details of Hot Water Immersion (HWI) Therapy. Percentages (%) and (N) values refer to the number of participants in the study who gave the corresponding answer.

**Did HWI help reduce the pain? (N=61)**	
**Yes**	77% (47)
No	6.6% (4)
Unsure/difficult to determine	16.4% (10)
**Did removal of the limb from the water cause the pain to return? (N=46)**	
Yes	70% (32)
No	30% (14)

**Table 2:** Data describing details of the effects on general life after a fish sting. N = 118 as some participants did not respond to this question. Percentages (%) and (N) values refer to the number of participants in the study who gave the corresponding answer.

**Was time taken off work (or school/college/university) due to the fish sting? (N = 119)**	
Yes	16 (14%) – mean no. days taken off 6 (±9)
No	102 (86%)
**Were any activities affected by pain at the sting site one week after the sting? (N = 58)**	
General movement of the affected limb	39 (84%)
Walking	7 (15%)
Work (incl. house chores)	11 (24%)
Sleep	10 (21%)
Hobbies/recreational activities	15 (32%)
Self-care	6 (13%)
General mood	8 (17%)
Socialising	2 (4%)

## Data Availability

The original contributions presented in this study are included in the article/Supplementary Material. Further inquiries can be directed to the corresponding authors.

## Supplementary Materials

The following supporting information can be downloaded at https://www.mdpi.com/article/10.3390/toxins17030134/s1. Table S1. Table summarising data for the country participants have been stung in; Table S2. Table summarising type of pain experienced according to groups of fishes; Table S3. Statistical output of paired t-test of the First vs Second sting data; Table S4. Table showing the pathophysiology of the sting wound/site; Table S5. Table highlighting the types of treatments people received and the uses of those treatments; Table S6. Table of known temperature range of HWI Therapy, if the temperature was tolerable, and if the temperature caused any further complications; Figure S1. Average pain rating across all groups of fishes. Error bars represent SEM; Figure S2. Descriptive statistics of pain ratings across different groups of fishes; Figure S3. Summary statistics of Male vs Female mixed effects model analyses in R; Figure S4. Summary statistics of Age Categories over time and Mixed effects model analyses in R; Figure S5. Summary statistics of Alive vs Dead mixed effects model analyses in R; Figure S6. Percentage distribution of individuals who reported an allergic reaction and/or infection after fish sting; Figure S7. Summary statistics of Treatment Categories mixed effects model analyses in R. Data S1. Data S1. Curated venomous fish sting data.

## Abbreviations

The following abbreviations are used in this manuscript:

DNIC	Diffuse Noxious Inhibitory Control
GC	Gating Control
HWI	How Water Immersion
IgE	Immunoglobulin E
NSAID	nonsteroidal anti-inflammatory drugs
SCUBA	Self-Contained Underwater Breathing Apparatus
