# Supplementary material for: Sting Stories: Firsthand Experiences of Fish Envenomation Through a Small-Scale Questionnaire
Source: Toxins (Basel). 2025 Mar 13;17(3):134. doi: 10.3390/toxins17030134 (PMC11946659; doi:10.3390/toxins17030134)
Supplement: Supplementary file 1 [file toxins-17-00134-s001.zip › toxins-3499416-supplementary.pdf]

toxins

MDPI

Supplementary Materials: Sting Stories: Firsthand Experiences of Fish Envenomation Through a Small-Scale Questionnaire

Richard J. Harris [1,*] , Silvia L. Saggiomo [2], Gillian Paxton [3] and Cherie A. Motti [1]

[1] Australian Institute of Marine Science (AIMS), Cape Cleveland, Townsville, QLD 4810, Australia
[2] Queensland Institute of Medical Research Berghofer (QIMR Berghofer), Brisbane, QLD 4006, Australia
[3] The Cairns Institute, James Cook University (JCU), Cairns, QLD 4870, Australia
* Correspondence: rharris2727@googlemail.com

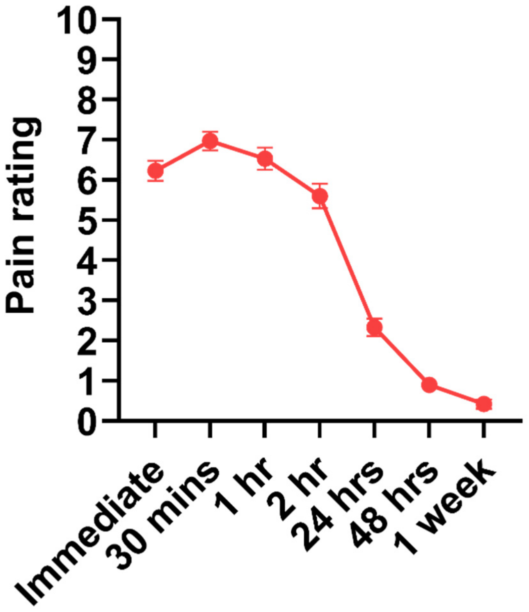

Figure S1. Average pain rating across all groups of fishes. Error bars represent SEM.

	Lionfish			Bullrout			Stonefish			Scorpionfish		
Immediate	5.731	2.947	67	8.75	1.5	4	7.5	3.507	6	6.833	2.401	6
30 min	6.866	2.486	67	9.25	0.9574	4	9.167	1.602	6	6.833	2.787	6
1 hr	6.701	2.995	67	8.75	0.9574	4	9	1.673	6	5.667	3.67	6
2 hr	5.836	3.301	67	7.25	1.708	4	8.667	2.066	6	4.833	3.971	6
24 hrs	2.493	2.351	67	1.5	3	4	4.5	3.391	6	2.167	2.401	6
48 hrs	0.7761	1.38	67	1	2	4	2	4	6	0.5	0.8367	6
1 week	0.2836	0.8129	67	0.25	0.5	4	2	4	6	0.3333	0.5164	6

	Weeverfish			Rabbitfish			Catfish			Stingrays		
Immediate	5.8	2.387	5	7.25	2.872	4	5.875	1.893	16	7.357	2.205	14
30 min	4	3.674	5	6.5	2.38	4	5.688	1.922	16	8.571	1.399	14
1 hr	2.6	3.647	5	5	3.559	4	4.938	2.435	16	8.071	1.639	14
2 hr	1.8	2.387	5	4	4.082	4	3.563	2.658	16	7.143	2.685	14
24 hrs	0.2	0.4472	5	0.75	0.9574	4	1.438	1.896	16	3.143	2.381	14
48 hrs	0	0	5	0	0	4	0.75	1.183	16	1.857	1.994	14
1 week	0	0	5	0	0	4	0.25	0.7746	16	0.9286	1.328	14

Figure S2. Descriptive statistics of pain ratings across different groups of fishes.

```
 Gender_pairwise Time_custom        estimate    SE  df lower.CL upper.CL t.ratio p.value
 Male - Female   Immediate.vs.1hr   -0.43697 0.551 824    -1.52    0.644  -0.793  0.4278
 Male - Female   Immediate.vs.1hr.1 -0.63866 0.551 824    -1.72    0.442  -1.160  0.2466
 Male - Female   Immediate.vs.2hr   -0.71092 0.551 824    -1.79    0.370  -1.291  0.1971
 Male - Female   Immediate.vs.24hrs -0.10252 0.551 824    -1.18    0.979  -0.186  0.8524
 Male - Female   Immediate.vs.48hrs -0.24538 0.551 824    -1.33    0.836  -0.446  0.6561
 Male - Female   Immediate.vs.1week -0.00672 0.551 824    -1.09    1.074  -0.012  0.9903

Confidence level used: 0.95
```

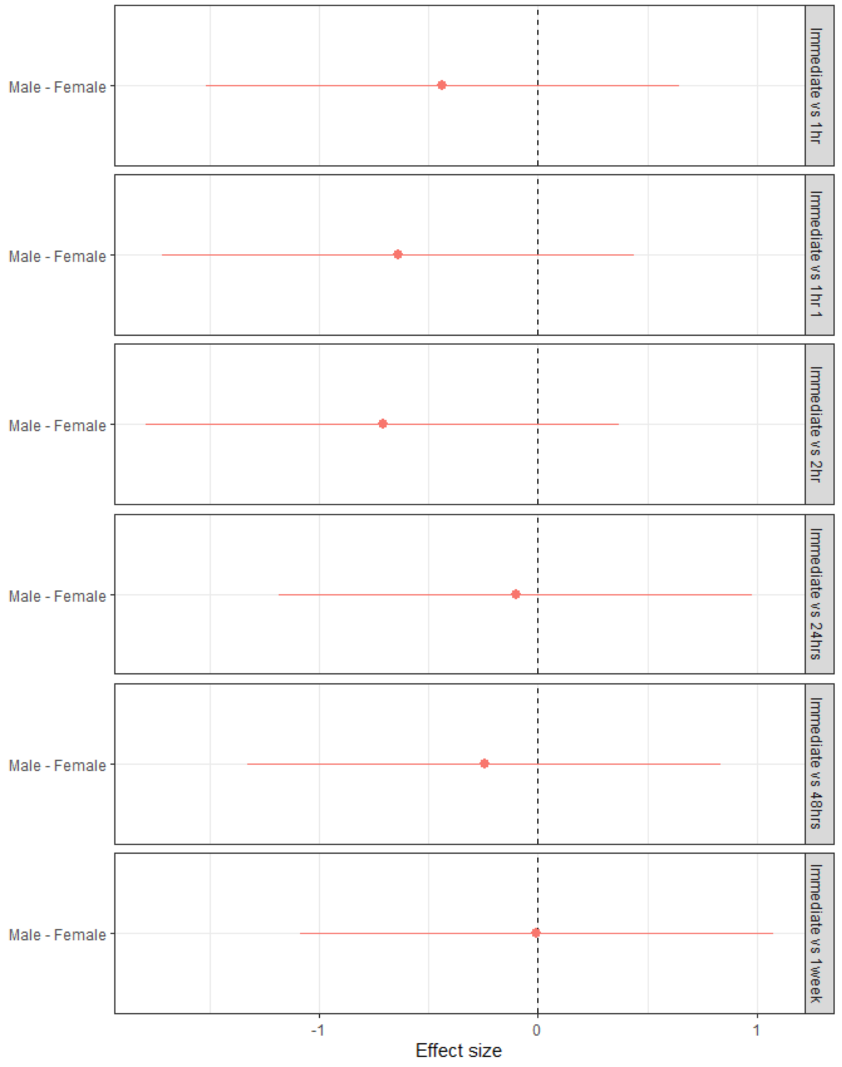

Figure S3. Summary statistics of Male vs Female mixed effects model analyses in R.

Treatment_pairwise	Time_custom	estimate	SE	df	lower.CL	upper.CL	t.ratio	p.value
Under 18 - (18-29)	Immediate.vs.30mins	-3.5037	1.320	789	-6.100	-0.9075	-2.649	0.0082
Under 18 - (30-39)	Immediate.vs.30mins	-2.7500	1.360	789	-5.416	-0.0837	-2.025	0.0432
Under 18 - (40-49)	Immediate.vs.30mins	-3.5273	1.350	789	-6.169	-0.8853	-2.621	0.0089
Under 18 - (50-59)	Immediate.vs.30mins	-3.0692	1.330	789	-5.673	-0.4652	-2.314	0.0209
Under 18 - (60-69)	Immediate.vs.30mins	-3.1333	1.370	789	-5.829	-0.4376	-2.282	0.0228
Under 18 - (70-79)	Immediate.vs.30mins	-0.3000	2.270	789	-4.762	4.1615	-0.132	0.8950
(18-29) - (30-39)	Immediate.vs.30mins	0.7537	0.801	789	-0.820	2.3269	0.940	0.3473
(18-29) - (40-49)	Immediate.vs.30mins	-0.0236	0.780	789	-1.555	1.5080	-0.030	0.9759
(18-29) - (50-59)	Immediate.vs.30mins	0.4345	0.746	789	-1.031	1.8997	0.582	0.5607
(18-29) - (60-69)	Immediate.vs.30mins	0.3704	0.827	789	-1.252	1.9930	0.448	0.6542
(18-29) - (70-79)	Immediate.vs.30mins	3.2037	1.990	789	-0.704	7.1115	1.609	0.1080
(30-39) - (40-49)	Immediate.vs.30mins	-0.7773	0.839	789	-2.425	0.8703	-0.926	0.3547
(30-39) - (50-59)	Immediate.vs.30mins	-0.3192	0.808	789	-1.905	1.2668	-0.395	0.6929
(30-39) - (60-69)	Immediate.vs.30mins	-0.3833	0.883	789	-2.116	1.3492	-0.434	0.6642
(30-39) - (70-79)	Immediate.vs.30mins	2.4500	2.010	789	-1.505	6.4047	1.216	0.2243
(40-49) - (50-59)	Immediate.vs.30mins	0.4580	0.787	789	-1.087	2.0028	0.582	0.5607
(40-49) - (60-69)	Immediate.vs.30mins	0.3939	0.863	789	-1.301	2.0887	0.456	0.6483
(40-49) - (70-79)	Immediate.vs.30mins	3.2273	2.010	789	-0.711	7.1656	1.609	0.1081
(50-59) - (60-69)	Immediate.vs.30mins	-0.0641	0.833	789	-1.699	1.5710	-0.077	0.9387
(50-59) - (70-79)	Immediate.vs.30mins	2.7692	1.990	789	-1.144	6.6823	1.389	0.1652
(60-69) - (70-79)	Immediate.vs.30mins	2.8333	2.020	789	-1.141	6.8080	1.399	0.1621
Under 18 - (18-29)	Immediate.vs.1hr	-3.3704	1.320	789	-5.967	-0.7741	-2.548	0.0110
Under 18 - (30-39)	Immediate.vs.1hr	-2.3000	1.360	789	-4.966	0.3663	-1.693	0.0908
Under 18 - (40-49)	Immediate.vs.1hr	-3.1818	1.350	789	-5.824	-0.5399	-2.364	0.0183
Under 18 - (50-59)	Immediate.vs.1hr	-2.5000	1.330	789	-5.104	0.1040	-1.885	0.0599
Under 18 - (60-69)	Immediate.vs.1hr	-2.7222	1.370	789	-5.418	-0.0265	-1.982	0.0478
Under 18 - (70-79)	Immediate.vs.1hr	2.5000	2.270	789	-1.962	6.9615	1.100	0.2717
(18-29) - (30-39)	Immediate.vs.1hr	1.0704	0.801	789	-0.503	2.6436	1.336	0.1821
(18-29) - (40-49)	Immediate.vs.1hr	0.1886	0.780	789	-1.343	1.7201	0.242	0.8091
(18-29) - (50-59)	Immediate.vs.1hr	0.8704	0.746	789	-0.595	2.3356	1.166	0.2439
(18-29) - (60-69)	Immediate.vs.1hr	0.6481	0.827	789	-0.974	2.2708	0.784	0.4332
(18-29) - (70-79)	Immediate.vs.1hr	5.8704	1.990	789	1.963	9.7782	2.949	0.0033
(30-39) - (40-49)	Immediate.vs.1hr	-0.8818	0.839	789	-2.529	0.7657	-1.051	0.2937
(30-39) - (50-59)	Immediate.vs.1hr	-0.2000	0.808	789	-1.786	1.3860	-0.248	0.8046
(30-39) - (60-69)	Immediate.vs.1hr	-0.4222	0.883	789	-2.155	1.3103	-0.478	0.6325
(30-39) - (70-79)	Immediate.vs.1hr	4.8000	2.010	789	0.845	8.7547	2.383	0.0174
(40-49) - (50-59)	Immediate.vs.1hr	0.6818	0.787	789	-0.863	2.2266	0.866	0.3865
(40-49) - (60-69)	Immediate.vs.1hr	0.4596	0.863	789	-1.235	2.1544	0.532	0.5947
(40-49) - (70-79)	Immediate.vs.1hr	5.6818	2.010	789	1.743	9.6202	2.832	0.0047
(50-59) - (60-69)	Immediate.vs.1hr	-0.2222	0.833	789	-1.857	1.4129	-0.267	0.7897
(50-59) - (70-79)	Immediate.vs.1hr	5.0000	1.990	789	1.087	8.9130	2.508	0.0123
(60-69) - (70-79)	Immediate.vs.1hr	5.2222	2.020	789	1.248	9.1969	2.579	0.0101
Under 18 - (18-29)	Immediate.vs.2hr	-3.6815	1.320	789	-6.278	-1.0853	-2.784	0.0055
Under 18 - (30-39)	Immediate.vs.2hr	-2.0000	1.360	789	-4.666	0.6663	-1.472	0.1413
Under 18 - (40-49)	Immediate.vs.2hr	-3.7909	1.350	789	-6.433	-1.1490	-2.817	0.0050
Under 18 - (50-59)	Immediate.vs.2hr	-2.4692	1.330	789	-5.073	0.1348	-1.861	0.0631
Under 18 - (60-69)	Immediate.vs.2hr	-2.5889	1.370	789	-5.285	0.1068	-1.885	0.0598
Under 18 - (70-79)	Immediate.vs.2hr	3.3000	2.270	789	-1.162	7.7615	1.452	0.1469
(18-29) - (30-39)	Immediate.vs.2hr	1.6815	0.801	789	0.108	3.2547	2.098	0.0362
(18-29) - (40-49)	Immediate.vs.2hr	-0.1094	0.780	789	-1.641	1.4222	-0.140	0.8885
(18-29) - (50-59)	Immediate.vs.2hr	1.2123	0.746	789	-0.253	2.6775	1.624	0.1048
(18-29) - (60-69)	Immediate.vs.2hr	1.0926	0.827	789	-0.530	2.7152	1.322	0.1866
(18-29) - (70-79)	Immediate.vs.2hr	6.9815	1.990	789	3.074	10.8893	3.507	0.0005

(30-39) - (40-49)	Immediate.vs.2hr	-1.7909	0.839	789	-3.438	-0.1434	-2.134	0.0332
(30-39) - (50-59)	Immediate.vs.2hr	-0.4692	0.808	789	-2.055	1.1168	-0.581	0.5616
(30-39) - (60-69)	Immediate.vs.2hr	-0.5889	0.883	789	-2.321	1.1436	-0.667	0.5048
(30-39) - (70-79)	Immediate.vs.2hr	5.3000	2.010	789	1.345	9.2547	2.631	0.0087
(40-49) - (50-59)	Immediate.vs.2hr	1.3217	0.787	789	-0.223	2.8664	1.680	0.0934
(40-49) - (60-69)	Immediate.vs.2hr	1.2020	0.863	789	-0.493	2.8968	1.392	0.1642
(40-49) - (70-79)	Immediate.vs.2hr	7.0909	2.010	789	3.153	11.0293	3.534	0.0004
(50-59) - (60-69)	Immediate.vs.2hr	-0.1197	0.833	789	-1.755	1.5154	-0.144	0.8858
(50-59) - (70-79)	Immediate.vs.2hr	5.7692	1.990	789	1.856	9.6823	2.894	0.0039
(60-69) - (70-79)	Immediate.vs.2hr	5.8889	2.020	789	1.914	9.8635	2.908	0.0037
Under 18 - (18-29)	Immediate.vs.24hrs	-1.1481	1.320	789	-3.744	1.4481	-0.868	0.3856
Under 18 - (30-39)	Immediate.vs.24hrs	0.2000	1.360	789	-2.466	2.8663	0.147	0.8830
Under 18 - (40-49)	Immediate.vs.24hrs	-1.2273	1.350	789	-3.869	1.4147	-0.912	0.3621
Under 18 - (50-59)	Immediate.vs.24hrs	-1.1154	1.330	789	-3.719	1.4886	-0.841	0.4007
Under 18 - (60-69)	Immediate.vs.24hrs	-1.3889	1.370	789	-4.085	1.3068	-1.011	0.3122
Under 18 - (70-79)	Immediate.vs.24hrs	3.5000	2.270	789	-0.962	7.9615	1.540	0.1240
(18-29) - (30-39)	Immediate.vs.24hrs	1.3481	0.801	789	-0.225	2.9214	1.682	0.0929
(18-29) - (40-49)	Immediate.vs.24hrs	-0.0791	0.780	789	-1.611	1.4525	-0.101	0.9192
(18-29) - (50-59)	Immediate.vs.24hrs	0.0328	0.746	789	-1.432	1.4980	0.044	0.9650
(18-29) - (60-69)	Immediate.vs.24hrs	-0.2407	0.827	789	-1.863	1.3819	-0.291	0.7709
(18-29) - (70-79)	Immediate.vs.24hrs	4.6481	1.990	789	0.740	8.5560	2.335	0.0198
(30-39) - (40-49)	Immediate.vs.24hrs	-1.4273	0.839	789	-3.075	0.2203	-1.701	0.0894
(30-39) - (50-59)	Immediate.vs.24hrs	-1.3154	0.808	789	-2.901	0.2706	-1.628	0.1039
(30-39) - (60-69)	Immediate.vs.24hrs	-1.5889	0.883	789	-3.321	0.1436	-1.800	0.0722
(30-39) - (70-79)	Immediate.vs.24hrs	3.3000	2.010	789	-0.655	7.2547	1.638	0.1018
(40-49) - (50-59)	Immediate.vs.24hrs	0.1119	0.787	789	-1.433	1.6566	0.142	0.8870
(40-49) - (60-69)	Immediate.vs.24hrs	-0.1616	0.863	789	-1.856	1.5332	-0.187	0.8516
(40-49) - (70-79)	Immediate.vs.24hrs	4.7273	2.010	789	0.789	8.6656	2.356	0.0187
(50-59) - (60-69)	Immediate.vs.24hrs	-0.2735	0.833	789	-1.909	1.3616	-0.328	0.7427
(50-59) - (70-79)	Immediate.vs.24hrs	4.6154	1.990	789	0.702	8.5284	2.315	0.0209
(60-69) - (70-79)	Immediate.vs.24hrs	4.8889	2.020	789	0.914	8.8635	2.414	0.0160
Under 18 - (18-29)	Immediate.vs.48hrs	-1.4296	1.320	789	-4.026	1.1666	-1.081	0.2801
Under 18 - (30-39)	Immediate.vs.48hrs	-0.3000	1.360	789	-2.966	2.3663	-0.221	0.8253
Under 18 - (40-49)	Immediate.vs.48hrs	-0.9818	1.350	789	-3.624	1.6601	-0.730	0.4659
Under 18 - (50-59)	Immediate.vs.48hrs	-1.0692	1.330	789	-3.673	1.5348	-0.806	0.4205
Under 18 - (60-69)	Immediate.vs.48hrs	-2.1333	1.370	789	-4.829	0.5624	-1.553	0.1207
Under 18 - (70-79)	Immediate.vs.48hrs	1.2000	2.270	789	-3.262	5.6615	0.528	0.5977
(18-29) - (30-39)	Immediate.vs.48hrs	1.1296	0.801	789	-0.444	2.7028	1.409	0.1591
(18-29) - (40-49)	Immediate.vs.48hrs	0.4478	0.780	789	-1.084	1.9794	0.574	0.5662
(18-29) - (50-59)	Immediate.vs.48hrs	0.3604	0.746	789	-1.105	1.8256	0.483	0.6294
(18-29) - (60-69)	Immediate.vs.48hrs	-0.7037	0.827	789	-2.326	0.9189	-0.851	0.3949
(18-29) - (70-79)	Immediate.vs.48hrs	2.6296	1.990	789	-1.278	6.5375	1.321	0.1869
(30-39) - (40-49)	Immediate.vs.48hrs	-0.6818	0.839	789	-2.329	0.9657	-0.812	0.4168
(30-39) - (50-59)	Immediate.vs.48hrs	-0.7692	0.808	789	-2.355	0.8168	-0.952	0.3414
(30-39) - (60-69)	Immediate.vs.48hrs	-1.8333	0.883	789	-3.566	-0.1008	-2.077	0.0381
(30-39) - (70-79)	Immediate.vs.48hrs	1.5000	2.010	789	-2.455	5.4547	0.745	0.4568
(40-49) - (50-59)	Immediate.vs.48hrs	-0.0874	0.787	789	-1.632	1.4573	-0.111	0.9116
(40-49) - (60-69)	Immediate.vs.48hrs	-1.1515	0.863	789	-2.846	0.5433	-1.334	0.1827
(40-49) - (70-79)	Immediate.vs.48hrs	2.1818	2.010	789	-1.757	6.1202	1.087	0.2772
(50-59) - (60-69)	Immediate.vs.48hrs	-1.0641	0.833	789	-2.699	0.5710	-1.277	0.2018
(50-59) - (70-79)	Immediate.vs.48hrs	2.2692	1.990	789	-1.644	6.1823	1.138	0.2553
(60-69) - (70-79)	Immediate.vs.48hrs	3.3333	2.020	789	-0.641	7.3080	1.646	0.1001
Under 18 - (18-29)	Immediate.vs.1week	-1.4370	1.320	789	-4.033	1.1592	-1.087	0.2776
Under 18 - (30-39)	Immediate.vs.1week	-0.8500	1.360	789	-3.516	1.8163	-0.626	0.5316
Under 18 - (40-49)	Immediate.vs.1week	-1.0818	1.350	789	-3.724	1.5601	-0.804	0.4218

Under 18 - (50-59)	Immediate.vs.1week	-1.0154	1.330	789	-3.619	1.5886	-0.765	0.4442
Under 18 - (60-69)	Immediate.vs.1week	-2.0111	1.370	789	-4.707	0.6846	-1.464	0.1435
Under 18 - (70-79)	Immediate.vs.1week	1.1000	2.270	789	-3.362	5.5615	0.484	0.6285
(18-29) - (30-39)	Immediate.vs.1week	0.5870	0.801	789	-0.986	2.1602	0.732	0.4641
(18-29) - (40-49)	Immediate.vs.1week	0.3552	0.780	789	-1.176	1.8868	0.455	0.6490
(18-29) - (50-59)	Immediate.vs.1week	0.4217	0.746	789	-1.044	1.8869	0.565	0.5723
(18-29) - (60-69)	Immediate.vs.1week	-0.5741	0.827	789	-2.197	1.0486	-0.694	0.4876
(18-29) - (70-79)	Immediate.vs.1week	2.5370	1.990	789	-1.371	6.4449	1.274	0.2029
(30-39) - (40-49)	Immediate.vs.1week	-0.2318	0.839	789	-1.879	1.4157	-0.276	0.7825
(30-39) - (50-59)	Immediate.vs.1week	-0.1654	0.808	789	-1.751	1.4206	-0.205	0.8379
(30-39) - (60-69)	Immediate.vs.1week	-1.1611	0.883	789	-2.894	0.5714	-1.316	0.1887
(30-39) - (70-79)	Immediate.vs.1week	1.9500	2.010	789	-2.005	5.9047	0.968	0.3334
(40-49) - (50-59)	Immediate.vs.1week	0.0664	0.787	789	-1.478	1.6112	0.084	0.9327
(40-49) - (60-69)	Immediate.vs.1week	-0.9293	0.863	789	-2.624	0.7655	-1.076	0.2821
(40-49) - (70-79)	Immediate.vs.1week	2.1818	2.010	789	-1.757	6.1202	1.087	0.2772
(50-59) - (60-69)	Immediate.vs.1week	-0.9957	0.833	789	-2.631	0.6393	-1.195	0.2323
(50-59) - (70-79)	Immediate.vs.1week	2.1154	1.990	789	-1.798	6.0284	1.061	0.2889
(60-69) - (70-79)	Immediate.vs.1week	3.1111	2.020	789	-0.864	7.0858	1.536	0.1248

Confidence level used: 0.95

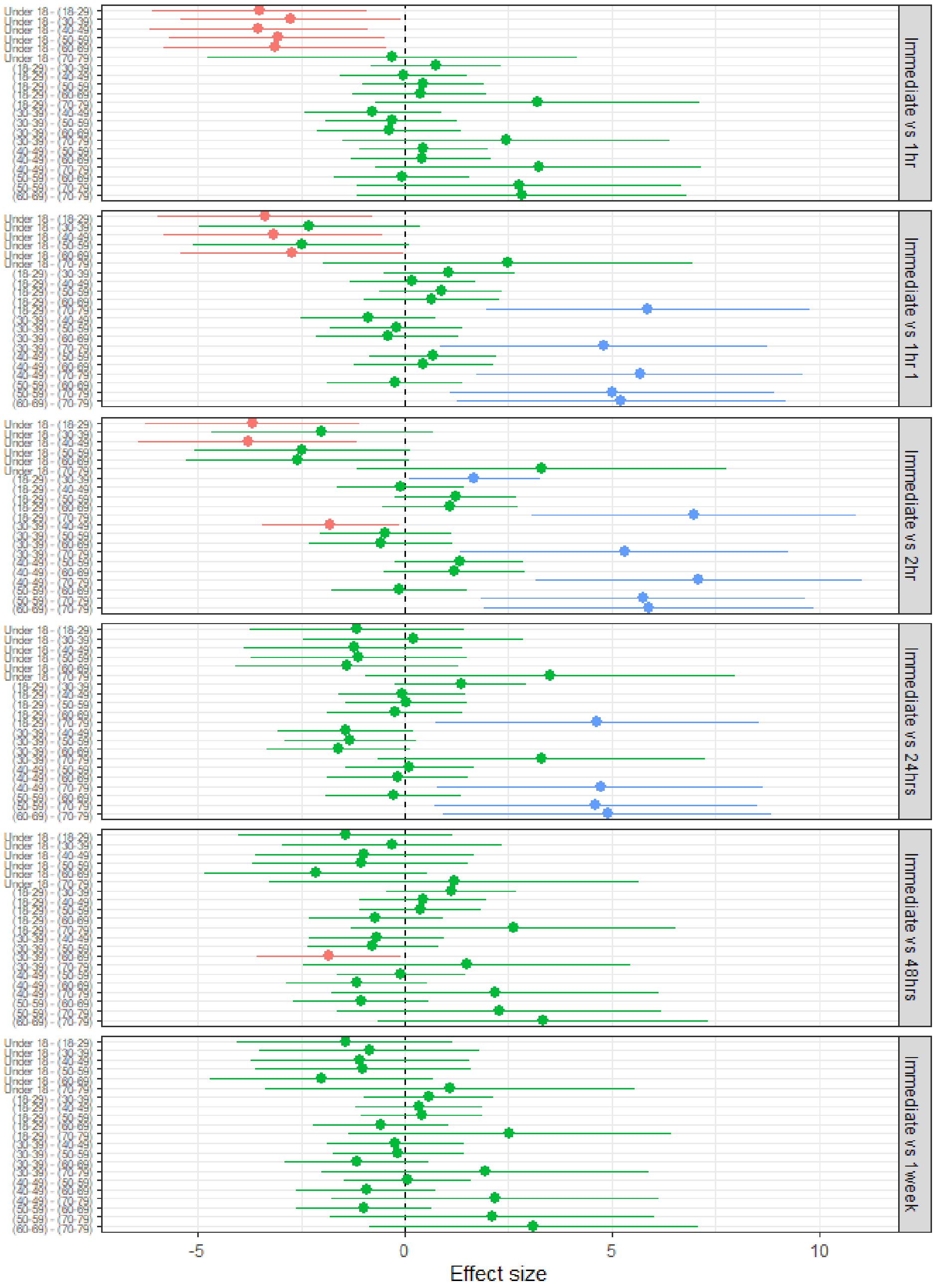

Figure S4. Summary statistics of Age Categories over time and Mixed effects model analyses in R.

Treatment_pairwise	Time_custom	estimate	SE	df	lower.CL	upper.CL	t.ratio	p.value
Alive - Dead	Immediately.vs.30mins	-2.150	0.94	824	-4.00	-0.304	-2.286	0.0225
Alive - Dead	Immediately.vs.1hr	-2.919	0.94	824	-4.76	-1.073	-3.104	0.0020
Alive - Dead	Immediately.vs.2hr	-3.276	0.94	824	-5.12	-1.430	-3.484	0.0005
Alive - Dead	Immediately.vs.24hrs	-1.733	0.94	824	-3.58	0.113	-1.843	0.0658
Alive - Dead	Immediately.vs.48hrs	-1.015	0.94	824	-2.86	0.831	-1.079	0.2808
Alive - Dead	Immediately.vs.1week	-0.853	0.94	824	-2.70	0.993	-0.907	0.3647

Confidence level used: 0.95

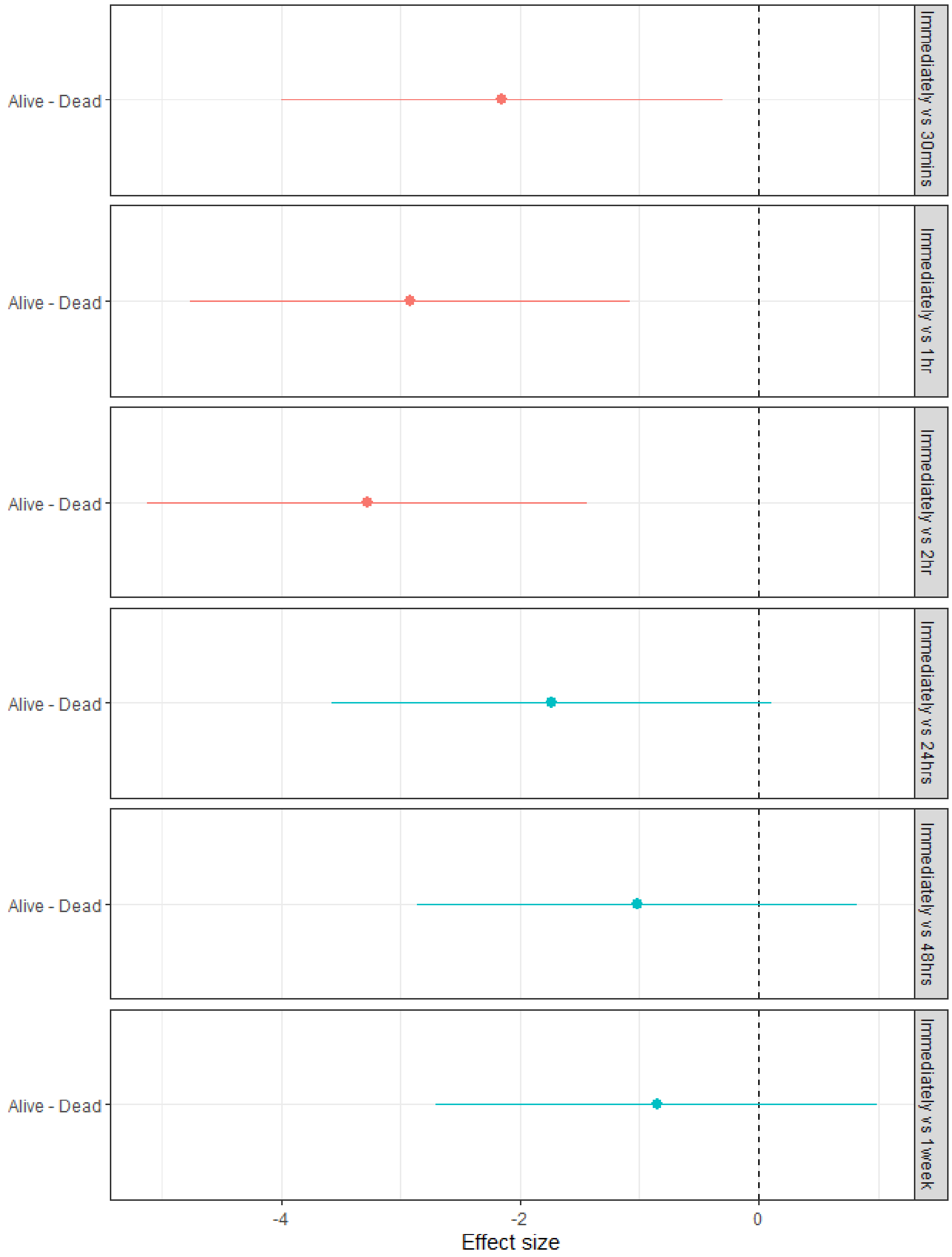

Figure S5. Summary statistics of Alive vs Dead mixed effects model analyses in R.

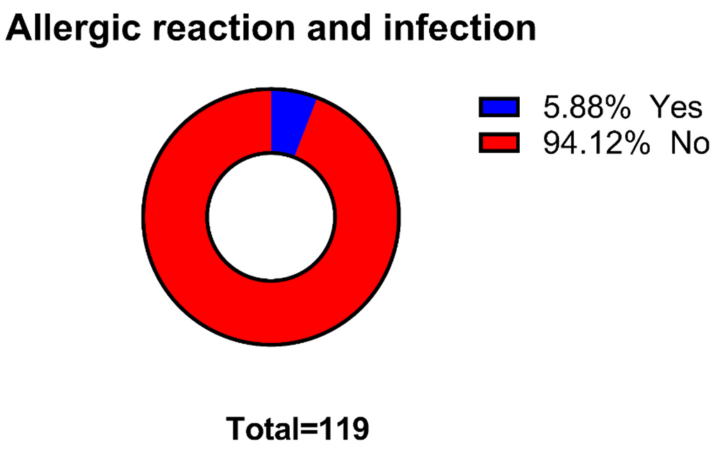

Figure S6. Percentage distribution of individuals who reported an allergic reaction and/or infection after fish sting.

Treatment_pairwise	Time_custom	estimate	SE	df	lower.CL	upper.CL	t.ratio	p.value
NT - T	X1hr.vs.2hr	-0.200	0.911	824	-1.989	1.5889	-0.219	0.8264
NT - HWI	X1hr.vs.2hr	-0.450	0.697	824	-1.818	0.9180	-0.646	0.5187
NT - (HWI+T)	X1hr.vs.2hr	0.302	0.549	824	-0.775	1.3796	0.551	0.5819
T - HWI	X1hr.vs.2hr	-0.250	1.020	824	-2.247	1.7467	-0.246	0.8059
T - (HWI+T)	X1hr.vs.2hr	0.502	0.922	824	-1.308	2.3123	0.545	0.5861
HWI - (HWI+T)	X1hr.vs.2hr	0.752	0.711	824	-0.643	2.1477	1.058	0.2902
NT - T	X1hr.vs.24hrs	-1.710	0.911	824	-3.499	0.0787	-1.876	0.0609
NT - HWI	X1hr.vs.24hrs	-0.860	0.697	824	-2.228	0.5078	-1.234	0.2175
NT - (HWI+T)	X1hr.vs.24hrs	-1.347	0.549	824	-2.425	-0.2701	-2.455	0.0143
T - HWI	X1hr.vs.24hrs	0.850	1.020	824	-1.147	2.8467	0.836	0.4036
T - (HWI+T)	X1hr.vs.24hrs	0.363	0.922	824	-1.447	2.1728	0.393	0.6941
HWI - (HWI+T)	X1hr.vs.24hrs	-0.487	0.711	824	-1.883	0.9082	-0.685	0.4933
NT - T	X1hr.vs.48hrs	-2.431	0.911	824	-4.220	-0.6417	-2.667	0.0078
NT - HWI	X1hr.vs.48hrs	-0.881	0.697	824	-2.249	0.4874	-1.264	0.2067
NT - (HWI+T)	X1hr.vs.48hrs	-2.531	0.549	824	-3.608	-1.4533	-4.611	<.0001
T - HWI	X1hr.vs.48hrs	1.550	1.020	824	-0.447	3.5467	1.524	0.1280
T - (HWI+T)	X1hr.vs.48hrs	-0.100	0.922	824	-1.910	1.7100	-0.108	0.9137
HWI - (HWI+T)	X1hr.vs.48hrs	-1.650	0.711	824	-3.045	-0.2546	-2.321	0.0205
NT - T	X1hr.vs.1week	-2.463	0.911	824	-4.252	-0.6743	-2.703	0.0070
NT - HWI	X1hr.vs.1week	-1.113	0.697	824	-2.481	0.2547	-1.597	0.1106
NT - (HWI+T)	X1hr.vs.1week	-2.791	0.549	824	-3.868	-1.7139	-5.086	<.0001
T - HWI	X1hr.vs.1week	1.350	1.020	824	-0.647	3.3467	1.327	0.1848
T - (HWI+T)	X1hr.vs.1week	-0.328	0.922	824	-2.138	1.4821	-0.356	0.7222
HWI - (HWI+T)	X1hr.vs.1week	-1.678	0.711	824	-3.073	-0.2825	-2.360	0.0185

Confidence level used: 0.95

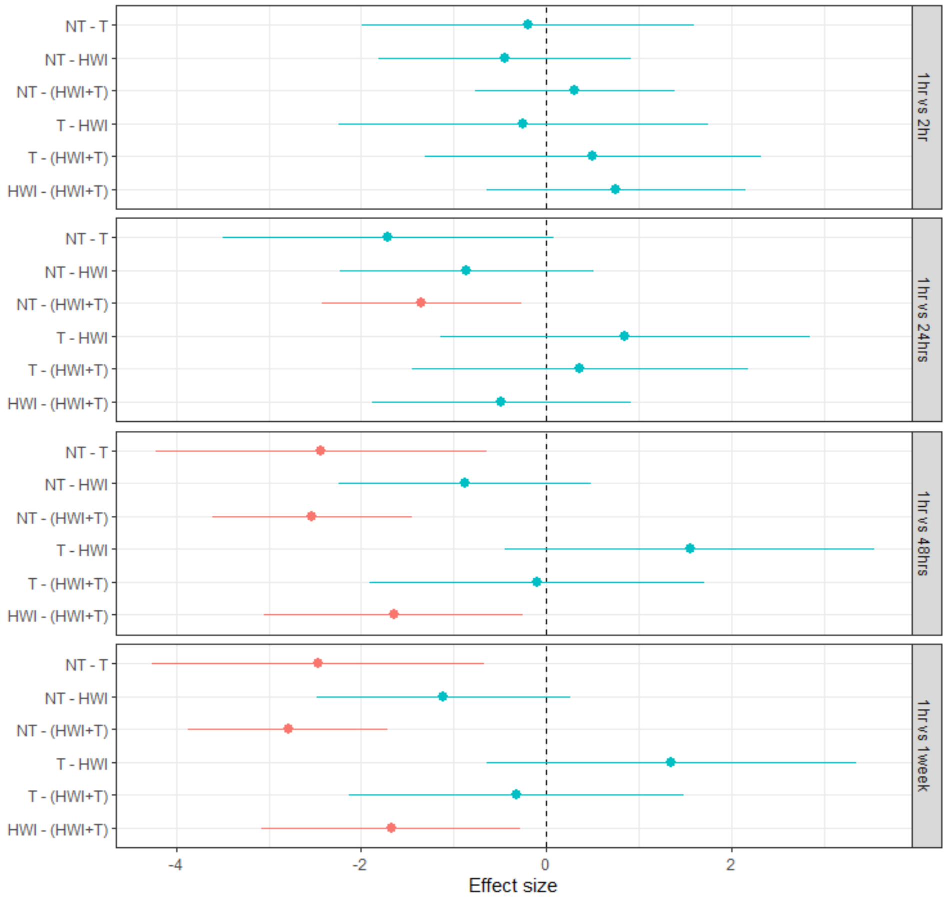

Figure S7. Summary statistics of Treatment Categories mixed effects model analyses in R.

Table S1. Table summarising data for the country participants have been stung in.

Country	Number of participants stung
Aruba	1
Australia	27
Bahamas	5
Belize	4
Bermuda	1
Bolivia	1
Bonaire	12
Brazil	3
Canada	1
Cayman Islands	1
Costa Rica	1
Cuba	2
Curacao	3
Dominica	4
France	1
Grand Cayman	1
Honduras	6
Jamaica	2
Mexico	3
Panama	2
Papua New Guinea	1
Puerto Rico	1
Roatan	1
Singapore	1
Taiwan	1
Thailand	2
Turks and Caicos	1
United Kingdom	6
US Virgin Islands	2
USA	38
Vanuatu	1

Table S2. Table summarising type of pain experienced according to groups of fishes.

	**Stonefish (*Synanceia* spp.)**	**Bullrout (*N. robusta*)**	**Lionfish (*Pterois* spp.)**	**Scorpionfish**	**Weeverfish (*E. vipera*)**	**Rabbitfish (*Siganus* spp.)**	**Catfish**	**Stingray**	**Combined**
Hot searing pain	6	4	46	4	4	3	11	10	**88 (83.5%)**
Cold pain	-	-	-	-	-	-	-	-	**0 (0%)**
Electric shock pain	-	-	3	-	-	-	-	1	**4 (3.9%)**
Pins and needles	-	-	3	1	-	1	1	-	**6 (5.8%)**
Itchiness	-	-	2	-	1	-	-	-	**3 (2.9%)**
Throbbing	-	-	2	-	-	-	1	1	**4 (3.9%)**

Table S3. Statistical output of paired t-test of the First vs Second sting data.

**Paired t test**	
P value	0.0112
P value summary	*
Significantly different (P < 0.05)?	Yes
One- or two-tailed P value?	Two-tailed
t, df	t=2.658, df=41
Number of pairs	42
How big is the difference?	
Mean of differences (A - B)	1.167
SD of differences	2.845
SEM of differences	0.4390
95% confidence interval	0.2801 to 2.053
R squared (partial eta squared)	0.1470
How effective was the pairing?	
Correlation coefficient (r)	0.2518
P value (one tailed)	0.0538
P value summary	ns
Was the pairing significantly effective?	No

Table S4. Table showing the pathophysiology of the sting wound/site.

	**Stonefish (*Synanceia* spp.)**	**Bullrout (*N. robusta*)**	**Lionfish (*Pterois* spp.)**	**Scorpionfish**	**Weeverfish (*E. vipera*)**	**Rabbitfish (*Siganus* spp.)**	**Catfish**	**Stingray**	**Combined**
Redness	4	3	46	4	2	3	9	10	**81 (32.2%)**
Paleness	-	-	1	1	1	-	-	1	**4 (1.9%)**
Swelling	3	3	54	5	2	3	-	9	**79 (37.3%)**
Bruise	-	1	8	1	-	-	3	4	**17 (8%)**
Necrosis	-	1	5	-	-	-	1	4	**11 (5.2%)**
Blister	1	1	12	-	-	-	2	-	**16 (7.5%)**
Anticoagulation	-	-	1	-	-	-	-	-	**1 (0.5%)**
Tingling	-	-	-	-	-	-	1	-	**1 (0.5%)**
Hardening of skin	-	-	2	-	-	-	-	-	**2 (0.9%)**

Table S5. Table highlighting the types of treatments people received and the uses of those treatments. An * implies these were the only pain-relieving treatments used as data for the HWI Therapy data analyses.

Other types of treatment used	N	Uses
Alcohol	3	Pain
Anaesthetics (local/inhalational)*	10	Pain
Antibiotics (unknown type)	1	Infection
Antivenom	3	Immunological
Benadryl	2	Antihistamine
Hot compress/heat pack	2	Pain
Ice	1	Pain
Pain medication/NSAIDs*	49	Pain
Tetanus vaccine	1	Infection
Urine/vinegar	2	Infection/pain

Table S6. Table of known temperature range of HWI Therapy, if the temperature was tolerable, and if the temperature caused any further complications.

Do you know what temperature the hot water was?	Was the temperature of the water tolerable?	Did the temperature of the water cause any more complications i.e., skin burns/scalding?
40-50°C (104-122°F)	Yes	No
40-50°C (104-122°F)	Yes	No
40-50°C (104-122°F)	Yes	No
50+°C (122+ °F)	Yes	No
Unknown	No	No
30-40°C (86-104°F)	Yes	No
30-40°C (86-104°F)	Yes	No
30-40°C (86-104°F)	Yes	No
Unknown	Yes	No
Unknown	Yes	No
Unknown	Yes	No
50+°C (122+ °F)	Yes	No
Unknown	Yes	No
Unknown	Yes	No

40-50°C (104-122°F)	Yes	No
40-50°C (104-122°F)	Yes	No
30-40°C (86-104°F)	Yes	No
Unknown	Yes	No
40-50°C (104-122°F)	Yes	No
40-50°C (104-122°F)	Yes	No
Unknown	No	No
Unknown	Yes	No
40-50°C (104-122°F)	No	No
30-40°C (86-104°F)	Yes	No
30-40°C (86-104°F)	Yes	No
Unknown	Yes	No
Unknown	Yes	No
40-50°C (104-122°F)	Yes	Yes
Unknown	No	Yes
50+°C (122+ °F)	Yes	Yes
Unknown	No	Yes
